# Circulating MicroRNAs Reflect Body Composition Features in Newly Diagnosed Breast Cancer

**DOI:** 10.3390/ijms27167098

**Published:** 2026-08-07

**Authors:** Federica Tambaro, Simona Orlando, Giovanni Imbimbo, Alessandro De Luca, Rossella Melcarne, Maria Ida Amabile, Maurizio Muscaritoli, Alessio Molfino

**Affiliations:** 1 Department of Translational and Precision Medicine, Sapienza University of Rome, 00185 Rome, Italy; federica.tambaro@uniroma1.it (F.T.); simona.orlando@uniroma1.it (S.O.); giovanni.imbimbo@uniroma1.it (G.I.); maurizio.muscaritoli@uniroma1.it (M.M.); 2 Department of Surgery, Sapienza University of Rome, 00185 Rome, Italy; dr.aless.deluca@gmail.com (A.D.L.); rossella.melcarne@uniroma1.it (R.M.); mariaida.amabile@uniroma1.it (M.I.A.); 3 Department of Interdisciplinary Studies on Wellbeing, Health and Environmental Sustainability (BeSSA), Sapienza University of Rome, 02100 Rieti, Italy

**Keywords:** breast cancer, body composition, microRNAs, miR-133a, muscle wasting, adipose tissue remodeling

## Abstract

Alterations in body composition are clinically relevant features occurring in patients with breast cancer (BC), but the mechanism remains poorly defined. We investigated whether circulating microRNAs (miRNAs) involved in skeletal muscle (SM) and adipose tissue (AT) metabolism are associated with body composition alterations in BC. We analyzed by RT-qPCR circulating levels of SM- and AT-related miRNAs in a cohort of breast cancer patients (BCPs) (*n* = 46) and controls (*n* = 16). Associations between miRNA profiles and body composition parameters were evaluated. miR-21, miR-29a, and miR-29b were upregulated in BCPs compared with controls (all *p* < 0.05). miR-133a was downregulated in patients with low muscle mass (*p* = 0.040), independently of adiposity. miR-133a was found to be lower in BCP subgroups with LUM-B and low levels of muscularity compared to those with LUM-A or LUM-B with high muscularity levels (all *p* < 0.05). Correlation analyses supported a closer association of miR-133a with SM-related parameters than with fat mass. miR-21, miR-29a and miR-29b appeared to reflect tumor-related systemic signaling. miR-133a was associated with reduced SM mass and influenced by tumor molecular subtype. These results suggest that miR-133a might represent a potential mediator of cancer-associated SM alterations and warrant validation in larger longitudinal studies.

## 1. Introduction

Breast cancer (BC) is the most diagnosed malignancy among women worldwide and a leading cause of cancer-related mortality, with incidence rates continuing to rise globally and an estimated annual increase of approximately 3.1% [1]. BC is characterized by substantial biological complexity, which extends beyond the tumor itself and contributes to systemic alterations affecting patient outcomes [2]. In addition to tumor-related burden, women diagnosed with BC frequently experience profound systemic alterations as a consequence of both the disease itself and antineoplastic treatments [3,4]. These include metabolic dysregulation, reduced energy expenditure, chronic inflammation, and decreased levels of physical activity. Collectively, these changes can significantly affect body composition, leading to adipose tissue (AT) remodeling and/or progressive loss of skeletal muscle (SM) mass [5]. Importantly, reductions in SM mass are already detectable at the time of diagnosis in more than one-third of newly diagnosed BC patients and have been independently associated with poorer clinical outcomes, including increased mortality risk [5]. Even more concerning is the frequent coexistence of loss of SM mass and functions (i.e., sarcopenia) with excess adiposity, a condition known as sarcopenic obesity (SO), in which quantitative or qualitative alterations in AT mask underlying SM wasting [6]. This condition represents a particularly detrimental phenotype, as it is associated with worse prognosis, reduced treatment tolerance, and significantly shorter progression-free survival compared with sarcopenia or obesity alone [7].

Despite increasing clinical recognition, the molecular mechanisms driving SM wasting and AT remodeling in early-stage BC remain poorly defined. Available evidence indicates that tumor-derived and systemic factors can disrupt SM and AT metabolism [8], suggesting the involvement of altered signaling pathways mediating inter-organ communication between the tumor and peripheral tissues. This complexity is further supported by the marked molecular heterogeneity of BC, with distinct tumor subtypes characterized by specific gene expression patterns and the activation of different signaling pathways that may differentially influence tumor progression and host metabolic adaptations [9].

At the molecular level, this systemic crosstalk is partly orchestrated by microRNAs (miRNAs), key post-transcriptional regulators capable of modulating metabolic, inflammatory, and catabolic pathways [10]. Aberrant miRNA expression has been extensively implicated in BC development and progression, supporting their role as key mediators of tumor biology and intercellular communication [11]. The dysregulation of specific miRNAs has been implicated in SM and AT metabolism [12,13], yet evidence linking circulating miRNA profiles to body composition changes in early-stage BC remains scarce.

In the present study, we aimed to characterize circulating miRNA signatures associated with early alterations in SM and AT and correlated their expression with body composition parameters observed in a cohort of newly diagnosed patients with BC and matched controls, to evaluate whether their differential modulation may unveil the underlying mechanism(s) of cancer-associated sarcopenia and sarcopenic obesity.

## 2. Results

### 2.1. Patient Characteristics

A total of forty-six BCPs and sixteen subjects undergoing surgery for non-malignant conditions were enrolled in this study. BCPs presented a mean age of 59.4 years, whereas the mean age for the control group was 49.3 years. Demographic, anthropometric and clinical characteristics are summarized in Table 1.

Among BCPs, the BMI mean was 26.8 kg/m^2^, whereas controls showed a mean of 23.5 kg/m^2^. Metabolic comorbidities were highly prevalent in the BCP cohort, with type 2 diabetes mellitus reported in 37%, dyslipidemia in 50%, metabolic syndrome in 50%, and hypertension in 54% of patients.

Regarding tumor characteristics, the most frequent molecular subtypes among the BCPs were luminal A (*n* = 18, 39%) and luminal B (*n* = 21, 46%), while triple-negative breast cancer (TNBC) accounted for 9% (*n* = 4) of cases and pathological carcinoma in situ (pTis) for 7% (*n* = 3).

### 2.2. Differences in Circulating miRNA Expression in Newly Diagnosed Breast Cancer Patients and Controls

To explore the involvement of circulating miRNAs in BC-related alterations of body composition, we selected a panel of miRNAs that we previously found to be implicated in both SM [14] and AT [12] metabolism. Their expression profiles were measured in plasma samples from BCPs and controls using RT-qPCR.

**Figure 1:**
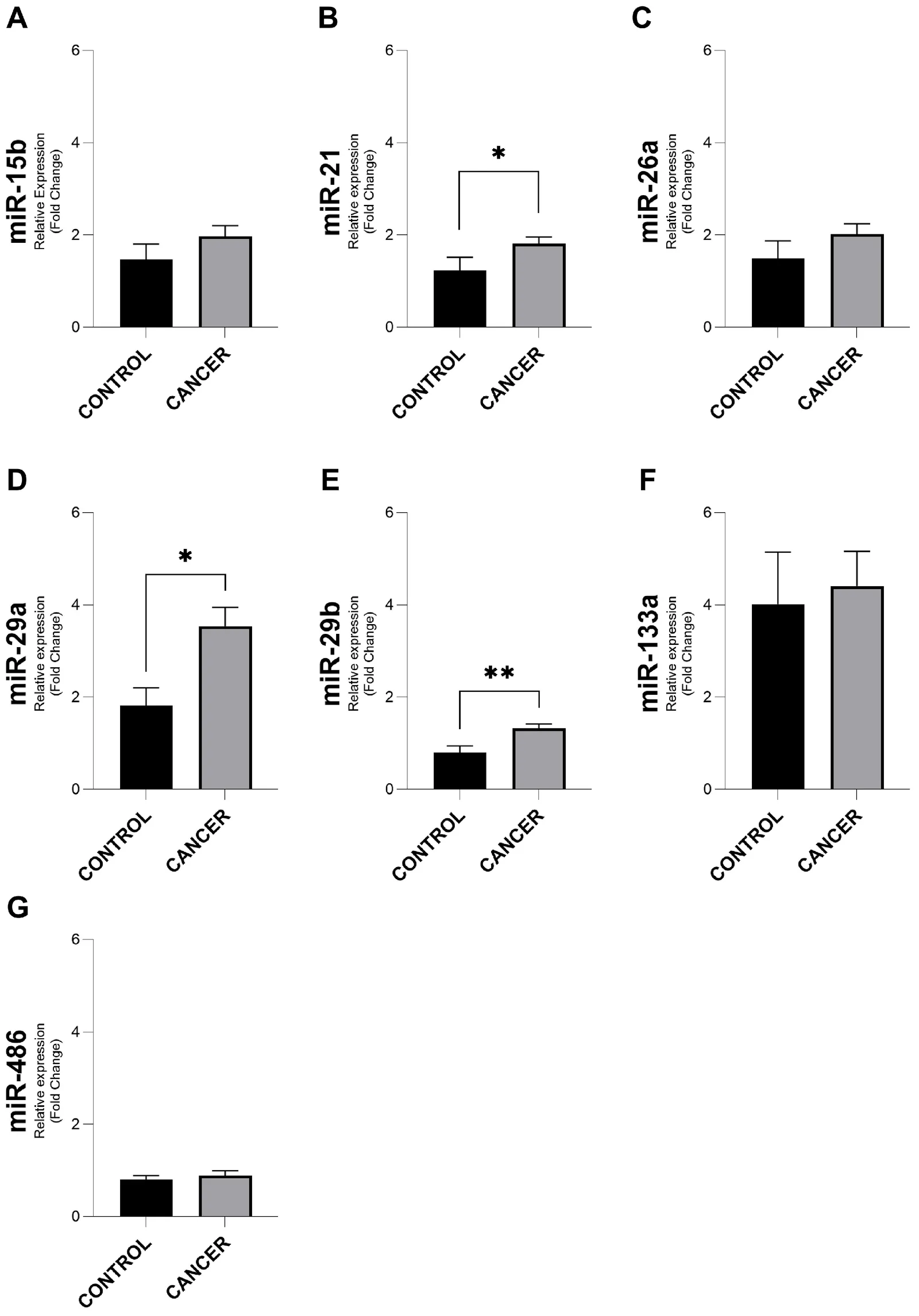
Differences in circulating miRNA expression in newly diagnosed breast cancer patients and controls. Circulating miRNA levels were analyzed by quantitative real-time PCR for patients with BC (*n* = 46) and the control group (*n* = 16). Data showed significantly higher expression levels of miR-21 (*p* = 0.043) (**B**), miR-29a (*p* = 0.014) (**D**), and miR-29b (*p* = 0.009) (**E**) in BCPs with respect to controls. No significant modulations were observed for miR-15b (**A**), miR-26a (**C**), miR-133a (**F**) or miR-486 (**G**). * *p* < 0.05, ** *p* ≤ 0.01.

BCPs exhibited a distinct circulating miRNA profile compared with controls (Figure 1), consisting in significant upregulation for miR-21 (*p* = 0.043), miR-29a (*p* = 0.014), and miR-29b (*p* = 0.009) in BCPs compared to controls (Figure 1B, Figure 1D and Figure 1E, respectively). No significant differences were detected for miR-15b, miR-26a, miR-133a or miR-486 when BCPs were compared with controls (Figure 1).

Given the established molecular heterogeneity of BC and its clinical relevance [15], we next evaluated circulating miRNA expression according to the molecular subtype. No significant differences in the expression of the analyzed miRNAs were detected in our cohort (Supplementary Figure S1).

**Figure 2:**
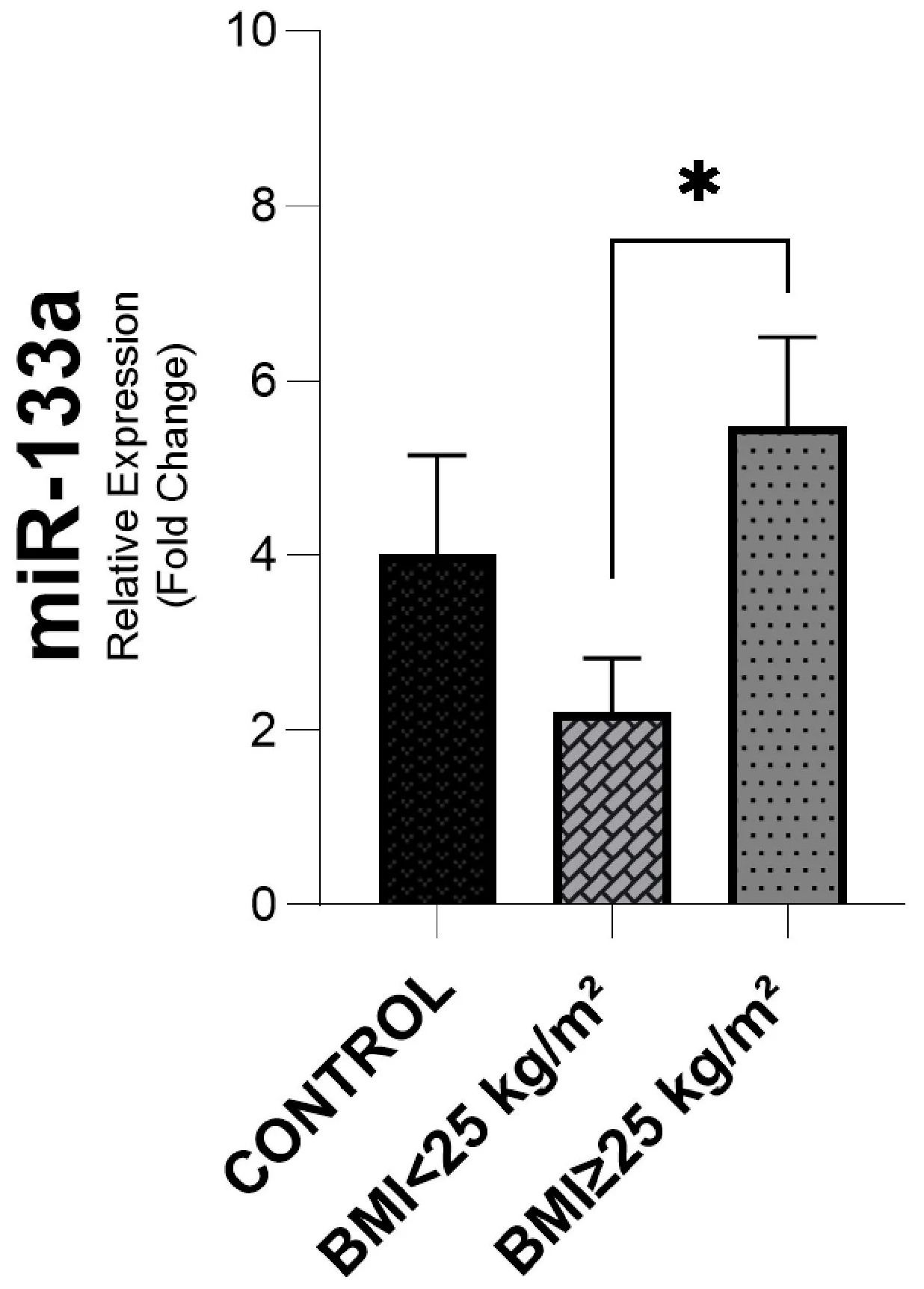
Differences in miRNA profile according to body mass index (BMI, kg/m^2^) categories. Circulating miRNA levels were analyzed according to different BMI categories, and BCPs were stratified considering BMI < 25 kg/m^2^ (normal weight) (*n* = 15) and BMI ≥ 25 kg/m^2^ (overweight and obese) (*n* = 31). Data show significantly lower expression of miR-133a in BCPs with BMI < 25 kg/m^2^ compared to those with BMI ≥ 25 kg/m^2^ (*p* = 0.021). * *p* < 0.05.

When participants were stratified into BMI < 25 kg/m^2^ (normal weight) and BMI ≥ 25 kg/m^2^ (overweight/obese) groups [16], a significant downregulation of miR-133a was observed in normal-weight BCPs compared with overweight/obese BCPs (*p* = 0.021) (Figure 2).

No additional significant differences in circulating miRNA expression were observed across these BMI categories (Supplementary Figure S2).

### 2.3. Different Modulation of Circulating miRNA According to Changes in Body Composition

To determine whether circulating miRNA modulation was associated with alterations in body composition, we next analyzed BIA-derived body composition parameters in BCPs, which are summarized in Table 2.

#### 2.3.1. Muscularity

Among the variables, FFMI was selected as the primary indicator of muscularity, given its established use as a normalized marker of SM mass [17]. This choice was further supported by the strong positive correlation between FFMI and ICW observed in our cohort (r = 0.990, *p* < 0.001). By using the median value of FFMI (17 kg/m^2^) as an internal cut-off, BCPs were stratified into low-muscle-mass and high-muscle-mass (LMM and HMM, respectively) groups.

**Figure 3:**
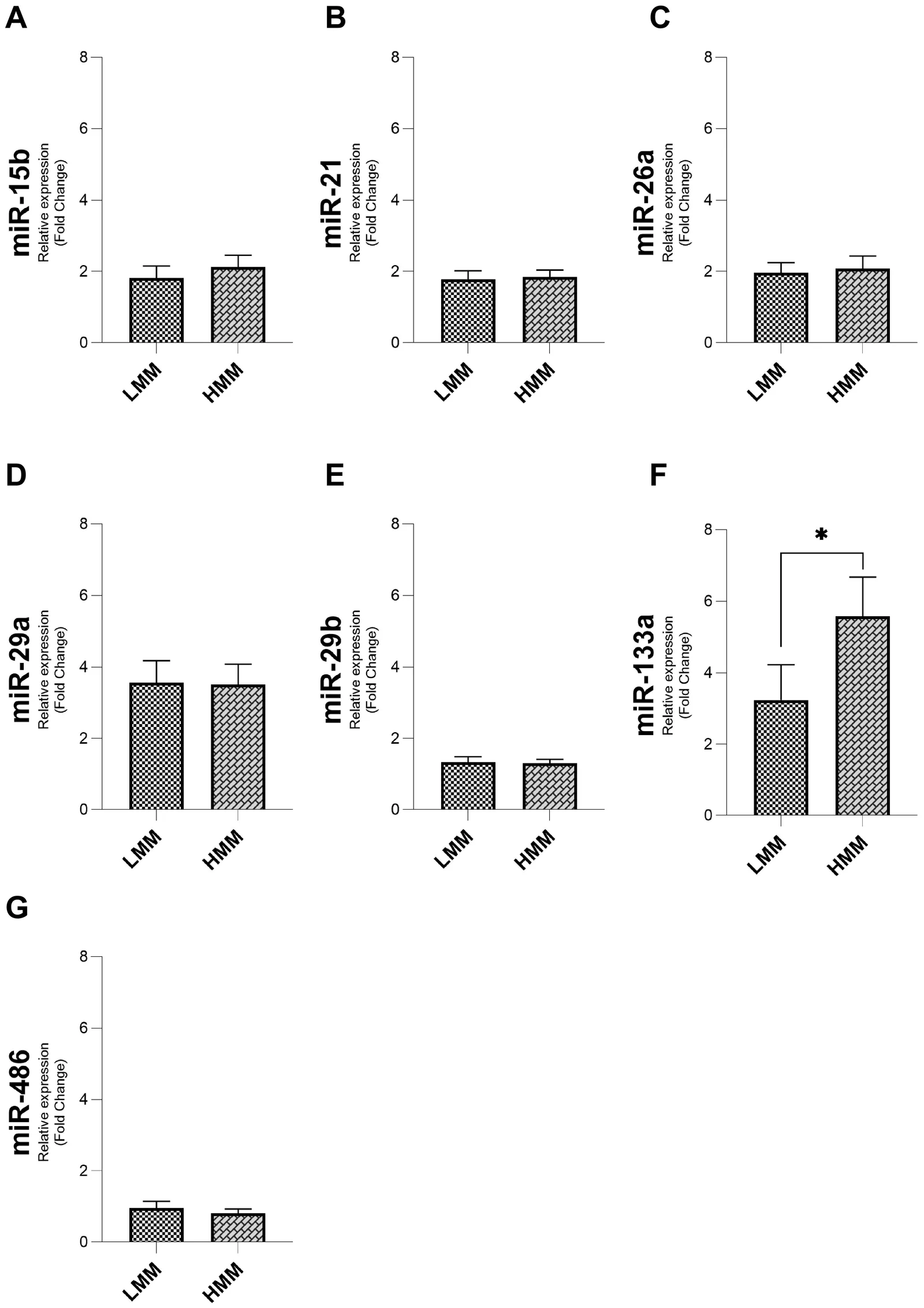
Different modulation of circulating miRNAs according to body composition. BCPs were stratified using the median value of FFMI (17 kg/m^2^) as an internal cut-off. Groups were the following: BCPs with LMM (*n* = 23) and BCPs with HMM (*n* = 23). (**F**) Data showed significantly lower levels of miR-133a (*p* = 0.040) in BCPs with LMM with respect to those with HMM. (**A**–**E**,**G**) No other significant modulation was observed for the other miRNAs. * *p* < 0.05.

As shown in Figure 3, miR-133a expression was significantly lower in BCPs with LMM compared with those with HMM (*p* = 0.040) (Figure 3F). No additional significant modulation was observed for the other circulating miRNAs when BCPs were stratified according to muscle mass levels (Figure 3).

**Figure 4:**
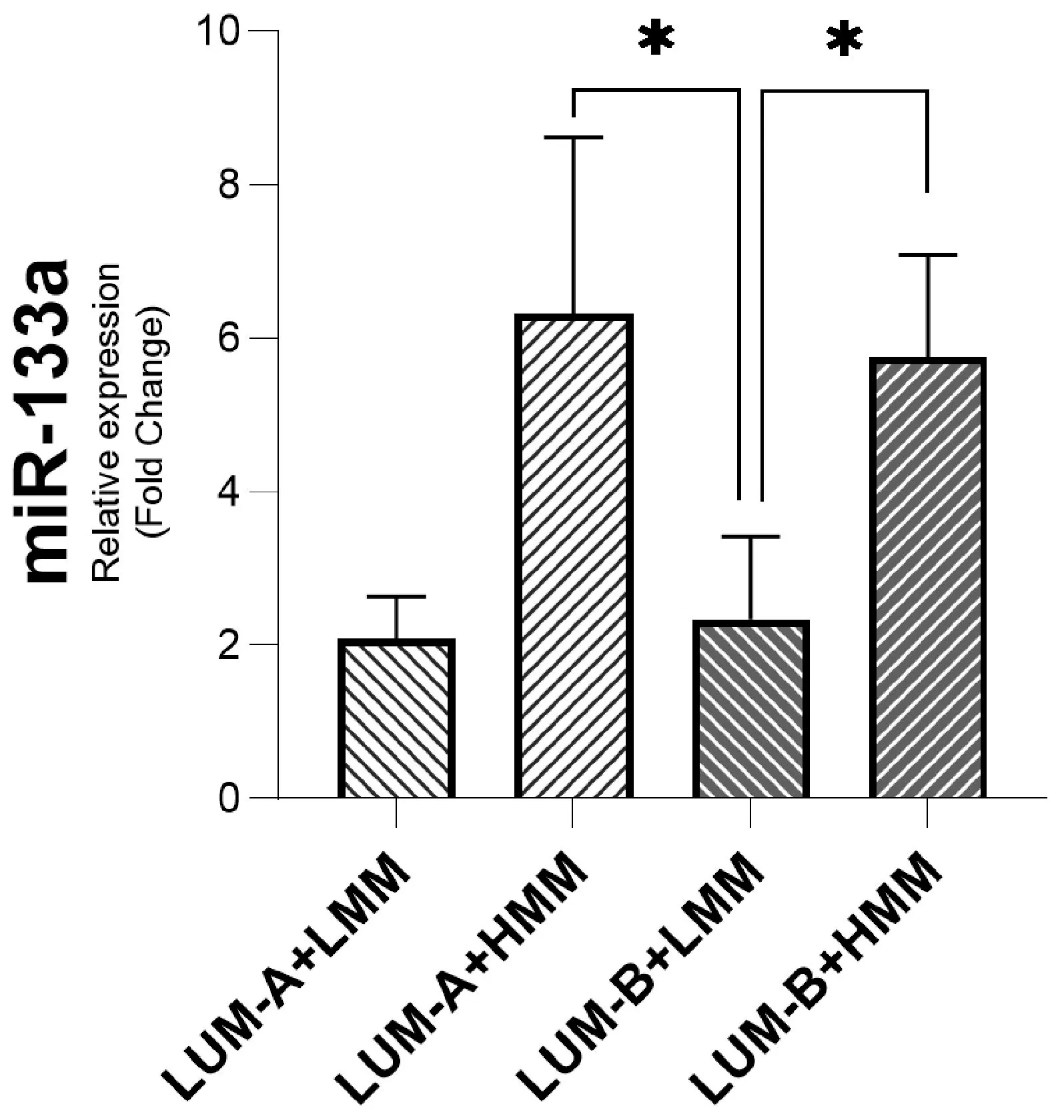
Differential expression profile of miR-133a in BCPs according to muscularity levels and tumor molecular subtypes. BCPs were stratified according to levels of muscularity and BC molecular subtypes (FFMI median cut-off value set at 17 kg/m^2^) as follows: LUM-A + LMM (*n* = 9); LUM-A + HMM (*n* = 8); LUM-B + LMM (*n* = 8); LUM-B + HMM (*n* = 13). miR-133a expression was higher in BCPs with LUM-A + HMM (*p* = 0.038) and LUM-B + HMM (*p* = 0.038) compared to those with LUM-B + LMM. * *p* < 0.05.

To assess whether this association was influenced by tumor-related factors, the level of muscularity was further examined in relation to molecular BC subtypes. We observed significantly lower expression of miR-133a in BCPs with luminal B (LUM-B) tumors and LMM compared to luminal A (LUM-A) patients with HMM (*p* = 0.038) and compared with those with LUM-B and HMM (*p* = 0.038) (Figure 4).

Additional differences in the expression of miR-21, miR-26a, and miR-29b were also observed for LUM-B patients with LMM versus HMM (*p* < 0.05) (Supplementary Figure S3).

#### 2.3.2. Adiposity

Given the high prevalence of AT alterations in early BC [18], we then evaluated circulating miRNA expression in relation to FMI. Using the median FMI value (9.9 kg/m^2^) as an internal threshold, patients were stratified according to lower or higher adiposity levels. Our results showed no significant modulation of circulating miRNA expression according to FMI levels (Supplementary Figure S4).

Finally, considering the clinical relevance of excess body weight in early breast cancer [19], we focused on BCPs with BMI ≥ 25 kg/m^2^ to explore the relationship between circulating miRNAs and body composition parameters. In this subgroup, miR-133a levels showed significant inverse correlations with FMI (r = −0.508, *p* = 0.004) and BF percentage (r = −0.565, *p* < 0.001), whereas no significant associations were observed with muscularity-related parameters (Table 3).

### 2.4. Modulation of Circulating miRNAs in Relation to Metabolic Comorbidities

**Figure 5:**
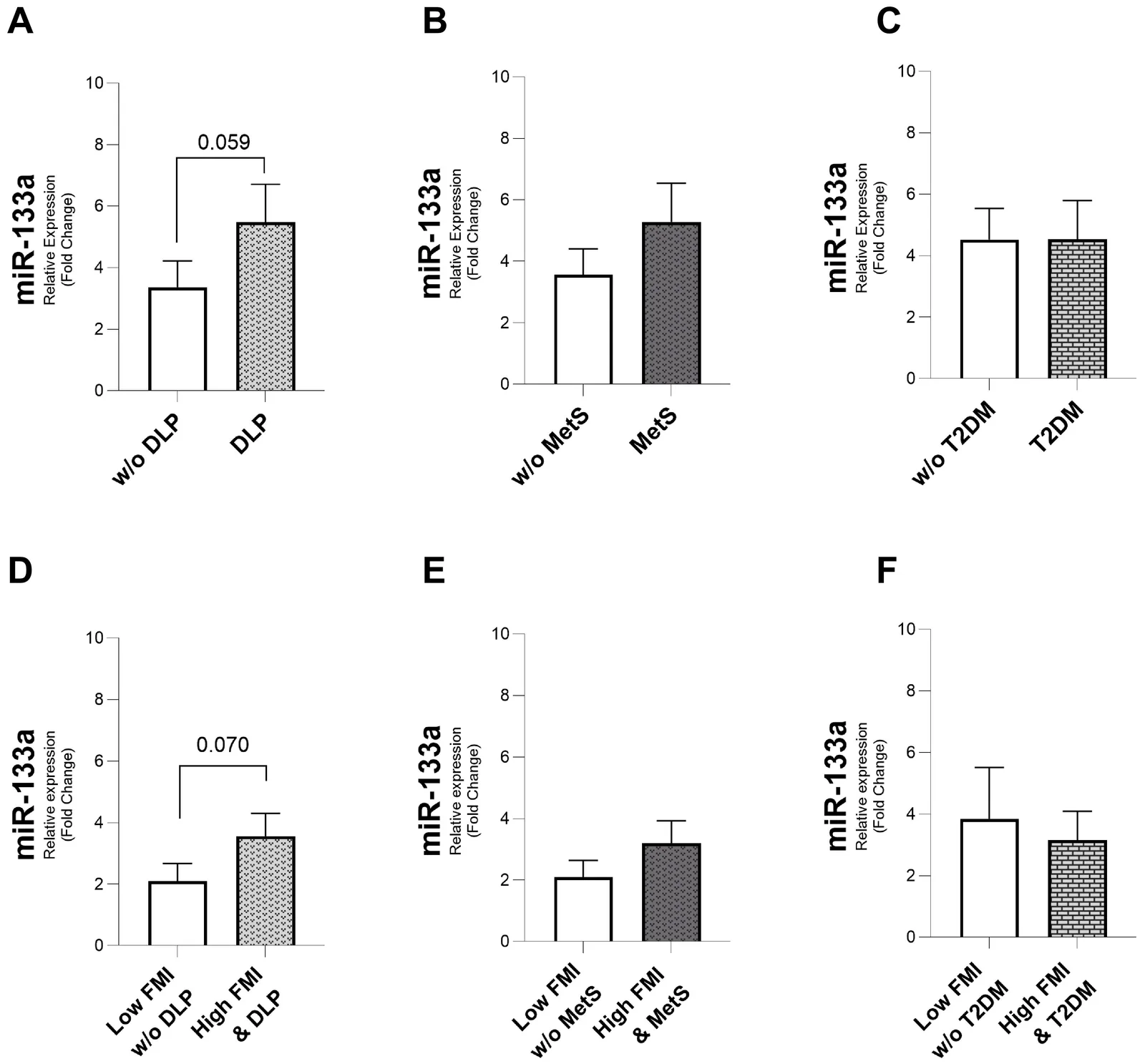
Modulation of circulating miRNAs according to body composition and presence of comorbidities. Circulating levels of miR-133a were analyzed considering the presence/absence of the main comorbidities and in combination with adiposity levels (internal FMI cut-off value set at 9.9 kg/m^2^). (**C**) A trend of modulation of mir-133a was observed according to the presence (*n* = 23) vs. absence (*n* = 22) of DLP (*p* = 0.059). (**A**,**B**) No significant modulation was observed by considering BCPs without (*n* = 27) or with (*n* = 17) T2DM, or without (*n* = 22) or with (*n* = 23) MetS. (**D**) A trend of modulation of miR-133a was observed in BCPs with low FMI without DLP (*n* = 14) vs. those with high FMI + DLP (*n* = 15) (*p* = 0.070). (**E**,**F**) No significant modulations were observed for BCP when the combined presence of T2DM or MetS with adiposity levels was considered.

Given the frequent coexistence of metabolic comorbidities in BC and their association with adverse clinical outcomes [20], we next evaluated whether the presence of major metabolic comorbidities could influence fat distribution and body composition alterations observed in BC through the modulation of circulating miRNAs (Figure 5).

When metabolic comorbidities were considered individually, we observed only a trend toward increased circulating levels of miR-133a in BCPs with DLP compared to those with no DLP (*p* = 0.059) (Figure 5C).

We did not observe a significant modulation of circulating levels of miR-133a according to the presence/absence of T2DM or MetS (Figure 5A,B). Similarly, no significant differences were observed for the other circulating miRNAs analyzed in relation to the presence of metabolic comorbidities.

We next explored the combined impact of metabolic comorbidities and adiposity, comparing BCPs without comorbidities and with low adiposity (FMI < 9.9 kg/m^2^) to those presenting metabolic comorbidities and higher FMI levels. A trend toward downregulation of circulating miR-133a levels was observed in BCPs with low FMI and without DLP compared to patients with high FMI and DLP (*p* = 0.070) (Figure 5F). No significant modulation of circulating miR-133a was detected when the combined presence of T2DM or MetS with adiposity levels was considered (Figure 5D,E). No significant differences were observed for the other circulating miRNAs analyzed when metabolic comorbidities were evaluated in combination with adiposity.

## 3. Discussion

In this cohort of newly diagnosed BCPs, circulating miRNA profiling revealed two distinct patterns. First, miR-21, miR-29a, and miR-29b were upregulated in patients compared with controls, supporting their association with tumor-related systemic signaling. Second, miR-133a emerged as the miRNA most closely associated with body composition, being significantly reduced in patients with low muscle mass and further modulated according to tumor molecular subtype. By contrast, adiposity and metabolic comorbidities exerted only limited effects on circulating miRNA expression. Although the investigated miRNAs have previously been associated with BC [21], our results indicate that they exhibit distinct relationships with body composition in the setting of BC naïve to treatment. These findings suggest that circulating miRNAs capture complementary aspects of tumor–host interactions, with miR-133a potentially reflecting skeletal muscle alterations that are present from the time of breast cancer diagnosis.

Of note, alterations in body composition are a clinically relevant yet still insufficiently characterized feature of BC. Emerging evidence indicates that changes in SM and AT may occur independently of overt weight loss or gain, reflecting early systemic adaptations to the tumor and host metabolic state [3]. These alterations are increasingly recognized as modulators of metabolic homeostasis, treatment tolerance, and clinical outcomes [5], although the molecular mechanisms linking BC to SM and AT remodeling remain incompletely understood. In this context, miRNAs involved in AT and SM homeostasis may represent key molecular mediators of BC-associated body composition alterations, integrating signals derived from tumor biology, peripheral tissues, and systemic metabolic regulation.

In the present study, we focused on a panel of circulating miRNAs with established roles in AT and SM metabolism, previously found to be deregulated in oncological settings [12,14]. We correlated their expression levels with BIA-derived body composition parameters in treatment-naïve early-stage BCPs and age-matched controls, aiming to uncover the biological mechanisms underlying BC-associated SM and AT alterations.

Overall, our findings underscore circulating miRNAs as integrative potential biomarkers of body composition remodeling in BC.

Among the miRNAs analyzed, circulating miR-15b levels exhibited no significant differences between BCPs and controls, nor were they associated with BIA-derived body composition parameters. Although miR-15b has been reported to play a role in metabolic processes including adipogenesis, lipid metabolism and muscle metabolism [22], in our cohort its circulating levels may primarily reflect intracellular or tissue-specific regulatory mechanisms rather than systemic body composition changes. Indeed, in the context of early-stage treatment-naïve BC, miR-15b modulation may therefore remain confined to local metabolic or tumor-related processes that are not readily captured at the circulating level.

MiR-21, one of the most extensively studied “oncomiRs”, showed significantly increased circulating levels in BCPs compared with controls. This finding is consistent with previous studies reporting elevated miR-21 levels in both the plasma and tumor tissue of BC patients [23,24]. In our cohort, however, circulating miR-21 levels were not significantly modulated when patients were stratified according to BMI, BIA-derived SM or AT parameters, tumor molecular subtype, or the presence of metabolic comorbidities. Considering its established roles in promoting tumor-associated inflammation, invasion, and fibrosis [25], and given the lack of correlations with body composition or metabolic factors, these findings suggest that circulating miR-21 upregulation may primarily reflect tumor-derived systemic signaling, largely independent of host SM and AT remodeling.

MiR-26a has been implicated in both cancer biology and metabolic regulation, including adipogenesis and muscle differentiation [26,27]. In our study, circulating miR-26a levels were not significantly modulated between BCPs and controls, nor were they associated with SM or AT parameters when considered independently. Similarly, no significant differences were observed when patients were stratified according to BIA-derived body composition parameters. However, when muscularity was analyzed in combination with tumor molecular subtype, a selective modulation of circulating miR-26a emerged, particularly in patients with low muscle mass and luminal B tumors. This context-specific pattern suggests that miR-26a deregulation may reflect intricate interactions between SM status and tumor features, positioning circulating miR-26a as a promising biomarker detectable only under specific combinations of host tissue remodeling and tumor subtype.

MiR-29a and miR-29b were both significantly upregulated in the circulation of BCPs compared with controls. This finding is consistent with previous reports describing increased circulating miR-29a levels in BC across disease stages [28] and with reports of miR-29b deregulation in primary BC tissues and cell lines, with functional data supporting anti-malignant effects upon its restoration [29]. In our cohort, neither miR-29a nor miR-29b varied significantly across BMI categories or with adiposity level, and no consistent modulation emerged when muscularity was considered alone. We speculate that their systemic upregulation may reflect qualitative remodeling processes, such as extracellular matrix reorganization or tumor–host signaling [30,31], rather than quantitative changes in fat or muscle mass.

MiR-486, a muscle-enriched miRNA pivotal for SM homeostasis, development, and function, has been implicated in BC-associated muscle dysregulation, with reports of decreased circulating levels in patients and tumor-bearing models [32,33]. In our cohort, however, circulating miR-486 levels exhibited no significant modulation between BCPs and controls, nor did they show any associations with BMI, BIA-derived parameters, or tumor molecular subtypes. This is in contrast with recent experimental evidence from murine BC models, where miR-486 overexpression protected against cancer-induced muscle loss, preserving contraction force, grip strength, and performance via the PTEN-AKT axis [34]. These findings suggest that miR-486-mediated regulatory mechanisms may predominantly operate at the tissue level and become evident in the circulation only in more advanced stages of skeletal muscle impairment, functional decline, or disease progression, remaining undetectable in early-stage, treatment-naïve breast cancer.

MiR-133a, a muscle-specific myomiR [10], was analyzed in relation to both tumor characteristics and host body composition parameters in our cohort. In line with previous reports [35,36], we did not observe significant modulation in circulating miR-133a levels between BCPs and controls. These findings suggest that, in early-stage treatment-naïve patients, circulating miR-133a is not primarily driven by tumor presence per se, prompting us to explore whether host-related factors could better explain its variability.

Overweight and obesity, defined by a BMI of 25–29.9 kg/m^2^ or ≥30 kg/m^2^, respectively, together with weight gain and body composition alterations, are increasingly recognized as clinically relevant factors in BC, influencing prognosis and treatment-related toxicity [37]. When BCPs were stratified according to BMI classes, we observed higher circulating miR-133a levels for those with BMI ≥ 25 kg/m^2^ compared with normal-weight BCPs. While previous studies have examined miR-133a according to BMI categories [38], BMI cannot distinguish between variations in skeletal muscle and adipose tissue. Consequently, the observed association with BMI may represent an indirect marker of underlying body composition differences, prompting us to further investigate the specific contribution of muscularity and adiposity. To better characterize the contribution of specific body components, we therefore extended our analysis by considering BIA-derived parameters in BCPs. When adiposity was evaluated using FMI, no significant modulation of circulating miR-133a was observed. In contrast, a significant downregulation emerged in patients with LMM, as defined by FFMI, independently of adiposity levels.

This pattern supports a preferential association between circulating miR-133a and SM status rather than fat mass expansion. Consistently, increased circulating miR-133a levels have been reported in BCPs classified as high responders to resistance exercise training [39]. Moreover, FFMI-related modulation of circulating miR-133a has also been described in patients with systemic sclerosis [40], further supporting a link between this myomiR and SM status across distinct pathological contexts.

To further clarify this association, linear correlation analyses were performed in the subgroup of BCPs with BMI ≥ 25 kg/m^2^, supporting a stronger relationship between miR-133a levels and SM parameters rather than adiposity indices. Importantly, the divergence between BMI- and FFMI-based stratifications suggests that the BMI-associated increase may be confounded by underlying differences in lean mass rather than reflecting adiposity per se.

Moreover, when FFMI was analyzed in combination with tumor molecular subtype, miR-133a levels were significantly reduced in LUM-B patients with LMM compared with patients with higher muscle mass and with LUM-A and LUM-B patients without LMM, suggesting a potential interaction between skeletal muscle status and tumor biological features. A comparable combined pattern was observed for miR-21 [24], miR-26a [41], and miR-29b [42]. However, since no significant modulation emerged when FFMI was analyzed independently, their modulation appears more consistent with tumor-intrinsic biological differences than with isolated SM alterations.

Given the proposed role of miRNAs at the interface between metabolic dysfunction and BC [43,44,45], we also examined whether circulating miR-133a levels varied according to metabolic comorbidity status. When patients were stratified according to the presence of major metabolic comorbidities, either alone or in combination with adiposity categories, only non-significant trends were observed. These findings suggest that metabolic comorbidities have a limited influence on circulating miR-133a levels at the time of BC diagnosis, whereas miR-133a modulation appears more closely associated with skeletal muscle status.

This study has several limitations. First, the relatively small sample size may have limited its statistical power, particularly in stratified analyses according to body composition parameters, tumor molecular subtypes, and metabolic comorbidities. Accordingly, some subgroup findings should be interpreted cautiously, given the limited number of patients in several subgroups, and considered exploratory until validated in larger, independent cohorts.

Second, body composition was assessed using BIA, which, although widely applied in clinical practice, does not provide the anatomical resolution or regional specificity of imaging-based techniques such as CT or DXA. Third, body composition was not assessed in the control group as part of the study protocol, precluding direct comparison of body composition parameters between breast cancer patients and controls. In addition, functional assessments of SM performance were not available, precluding direct evaluation of the relationship between circulating miR-133a and muscle function.

Finally, the observational and cross-sectional design precludes causal inference. Moreover, SM function was not assessed using objective measures such as handgrip strength or physical performance tests, precluding evaluation of the relationships between circulating miRNAs, SM mass, and SM function. For this reason, we cannot determine whether circulating miR-133a modulation contributes to body composition alterations or instead reflects underlying SM remodeling processes. Longitudinal and mechanistic studies will be required to clarify the relative contribution of tumor-intrinsic factors, host metabolic status, treatment exposure, and multi-organ crosstalk in shaping circulating miRNA profiles in breast cancer.

## 4. Materials and Methods

### 4.1. Study Design and Patient Enrollment

This observational, monocentric study included consecutive patients with newly diagnosed, histologically confirmed primary breast cancer who were referred to the Department of Surgery (formerly Department of Surgical Sciences), Sapienza University of Rome, Italy. All experimental procedures were conducted at the Department of Translational and Precision Medicine within the same institution.

Patients were enrolled at the time of diagnosis, prior to any anticancer treatment, and were selected based on eligibility for surgical tumor resection with curative intent. The inclusion criteria were age ≥ 18 years, eligibility for surgery with curative purposes, and absence of prior exposure to anticancer, corticosteroid, or anti-inflammatory treatments and the concomitant presence of catabolic conditions. The exclusion criterion was any condition precluding informed consent, including refusal to participate in the study.

The control group consisted of women undergoing surgery for benign, non-malignant conditions, with no history of cancer or chronic inflammatory diseases.

The study was conducted in accordance with the Declaration of Helsinki and approved by the local Ethics Committee (Sapienza University, Azienda Policlinico Umberto I, Rome, Italy—prot. n. 895/18).

All participants provided written informed consent prior to study participation.

### 4.2. Clinical and Nutritional Assessment and Body Composition Analysis

Clinical assessments, demographic characteristics, and medical histories, including comorbidities such as metabolic syndrome (MetS), dyslipidemia (DLP), and type 2 diabetes mellitus (T2DM), as well as information on tumor histology and molecular breast cancer subtypes, were recorded for all participants at the time of enrollment.

Anthropometric measurements, including body weight (kg) and height (m), were used to calculate the body mass index (BMI, kg/m^2^).

Fasting peripheral blood samples were collected in EDTA Vacutainer^®^ (Becton, Dickinson and Company, Franklin Lakes, USA) tubes and centrifuged at 3000 rpm for 15 min at 4 °C. Plasma was immediately aliquoted into cryovials and stored at −80 °C until further analysis.

Bioelectrical impedance analysis (BIA) was performed in all BCPs before surgery, using a research-grade, multifrequency body composition analyzer applying an alternating electric current of 800 μA at 50 Hz frequency (InBody 770^®^, InBody Co., Ltd., Caresmed, Milan, Italy).

The fat-free mass index (FFMI, kg/m^2^), phase angle (PhA, °), fat mass index (FMI, kg/m^2^), body fat percentage (BF, %), and intracellular water (ICW, L) were obtained using the manufacturer-provided software (LookinBody120, Seoul, Republic of Korea).

### 4.3. Extraction, Quantification, and Analysis of Circulating miRNAs

Total RNA was extracted from plasma samples using the RNeasy Serum/Plasma Kit (Qiagen, Germantown, MD, USA) according to the manufacturer’s instructions. RNA quantification was performed using a Multiskan Sky spectrophotometer (Thermo Fisher Scientific, Waltham, MA, USA). cDNA was synthesized from 10 ng of total RNA using the TaqMan Advanced miRNA cDNA Synthesis kit (Applied Biosystems, Thermo Fisher Scientific, Waltham, MA, USA) following the manufacturer’s instructions. Quantitative real-time PCR was performed with TaqMan Fast Advanced Master Mix (Applied Biosystems, Thermo Fisher Scientific, Waltham, MA, USA), using the QuantStudio^®^ Real Time PCR (Applied Biosystems, Thermo Fisher Scientific, Waltham, MA, USA). Quantification was performed using the specific TaqMan miRNA assay probe (A25576, Applied Biosystems, Thermo Fisher Scientific, Waltham, MA, USA): hsa-miR-15b-5p, hsa-miR-21-5p, hsa-miR-26a-5p, hsa-miR-29a-3p, hsa-miR-29b-3p, hsa-miR-133a-3p, and hsa-miR-486-5p.

The expression of hsa-miR-16-5p was used as the endogenous control to normalize the sample data replicates, and a calibrator sample was used as an internal control. The resulting data were analyzed using SDS4.4 Software (Applied Biosystems, Bedford, MA, USA), and relative expression levels were calculated using the 2^−ΔΔCt^ method. All experiments were performed in duplicate.

### 4.4. Statistical Analyses

Data are presented as the mean ± standard deviation (SD) or standard error of the mean (SEM) and median with interquartile range (IQR) (25th and 75th percentile) for continuous normally and non-normally distributed variables, as appropriate. Student’s *t*-test and one-way ANOVA or the Mann–Whitney test and Kruskal–Wallis test were used for normally distributed data or non-normally distributed data, respectively.

Spearman’s correlation was used for correlation analyses of miRNAs and body composition parameters. A *p*-value < 0.05 was considered statistically significant. Statistical analyses were performed using IBM^®^ SPSS Statistics version 30.0 (SPSS Inc., Chicago, IL, USA) and GraphPad Prism 10.2.3 (San Diego, CA, USA).

## 5. Conclusions

Our findings indicate that circulating miR-133a in early-stage, treatment-naïve breast cancer patients may be more closely associated with SM status than with adiposity or overt metabolic comorbidities. The significant downregulation observed in patients with low SM mass, particularly within the LUM-B subtype, suggests a potential interplay between host SM condition and tumor biology. These data highlight the importance of integrating circulating miRNA profiling with refined body composition assessment to improve patient stratification. Overall, our results suggest that circulating miR-133a may represent a promising indicator of SM alterations in early-stage breast cancer and further provide additional support for the concept that circulating miRNAs can capture clinically relevant aspects of tumor–host interactions in breast cancer.

## Figures and Tables

**Table 1:** Clinical and demographic characteristics of the study participants.

	Controls (*n* = 16)	Patients with Breast Cancer (*n* = 46)	*p*-Value
Age (years), mean ± SD	49.3 ± 13.0	59.4 ± 14.2	0.007 *
Actual weight (kg), mean ± SD	62.9 ± 10.0	70.9 ± 13.0	0.014 *
BMI (kg/m^2^), mean ± SD	23.5 ± 3.9	26.8 ± 4.3	0.004 *
Blood parameters:			
Creatinine (mg/dL), mean ± SD	0.77 ± 0.1	0.82 ± 0.4	0.424
Albumin (g/dL), mean ± SD	40.0 ± 2.8	45.2 ± 5.8	0.061
BUN (mmol/L), mean ± SD	10.5 ± 2.8	9.4 ± 4.7	0.123
Glycaemia (mmol/L), mean ± SD	5.2 ± 1.3	5.7 ± 1.0	0.546
Total cholesterol (mg/dL), mean ± SD	171.5 ± 34.7	198.0 ± 43.1	0.741
Triglycerides (mmol/L), mean ± SD	95.5 ± 3.5	112.8 ± 39.1	0.647
Main comorbidities:			
Diabetes mellitus, *n* (%)	/	17 (37)	
Dyslipidemia, *n* (%)	/	23 (50)	
Metabolic syndrome, *n* (%)	/	23 (50)	
Hypertension, *n* (%)	/	25 (54)	
Molecular subtype, *n* (%):			-
Luminal A	/	18 (39)	
Luminal B	/	21 (46)	
TNBC	/	4 (9)	
pTis	/	3 (7)	

Abbreviations: BMI, body mass index; BUN, Blood Urea Nitrogen; TNBC, triple-negative breast cancer; pTis, pathological tumor in situ; SD, standard deviation. * indicates statistically significant *p*-values.

**Table 2:** Body composition parameters of newly diagnosed breast cancer patients assessed by bioelectrical impedance analysis.

Body Composition Parameters	Breast Cancer Patients (*n* = 46)
FFMI (kg/m^2^), mean ± SD	16.9 ± 0.2
FMI (kg/m^2^), mean ± SD	9.9 ± 0.5
ICW (L), mean ± SD	20 ± 0.4
BF (%), median (IQR)	37.1 (32; 41)
PhA (°), mean ± SD	4.4 ± 0.1

Abbreviations: FFMI, fat-free mass index; FMI, fat mass index; ICW, intracellular water; BF, body fat; PhA, phase angle; SD, standard deviation; IQR, interquartile range.

**Table 3:** Spearman’s correlation analysis of miR-133a with BIA-derived parameters in breast cancer patients with BMI ≥ 25 kg/m^2^.

	miR-133a (*n* = 31)
FMI, kg/m^2^	*r* = −0.508 *p* = 0.004
FFMI, kg/m^2^	*r* = −0.081 *p* = 0.666
PhA, °	*r* = 0.013 *p* = 0.945

## Data Availability

The data that support the findings of this study are available from the corresponding author upon reasonable request.

## Supplementary Materials

The following supporting information can be downloaded at https://www.mdpi.com/article/10.3390/ijms27167098/s1.

## Abbreviations

The following abbreviations are used in this manuscript:

AT	Adipose tissue
BC	Breast cancer
BCP	Breast cancer patient
BF	Body fat
BIA	Bioelectrical impedance analysis
BMI	Body mass index
DLP	Dyslipidemia
FFMI	Fat-free mass index
FMI	Fat mass index
HMM	High muscle mass
ICW	Intracellular water
LMM	Low muscle mass
LUM-A	Luminal A
LUM-B	Luminal B
MetS	Metabolic syndrome
miRNA/miR	microRNA
PhA	Phase angle
SM	Skeletal muscle
SO	Sarcopenic obesity
T2DM	Type 2 diabetes mellitus
