# Supplementary material for: Circulating MicroRNAs Reflect Body Composition Features in Newly Diagnosed Breast Cancer
Source: Int J Mol Sci. 2026 Aug 7;27(16):7098. doi: 10.3390/ijms27167098 (PMC13513458; doi:10.3390/ijms27167098)
Supplement: Supplementary file 1 [file ijms-27-07098-s001.zip › ijms-4465969-supplementary final 2.0.pdf]

Supplementary Figure S1

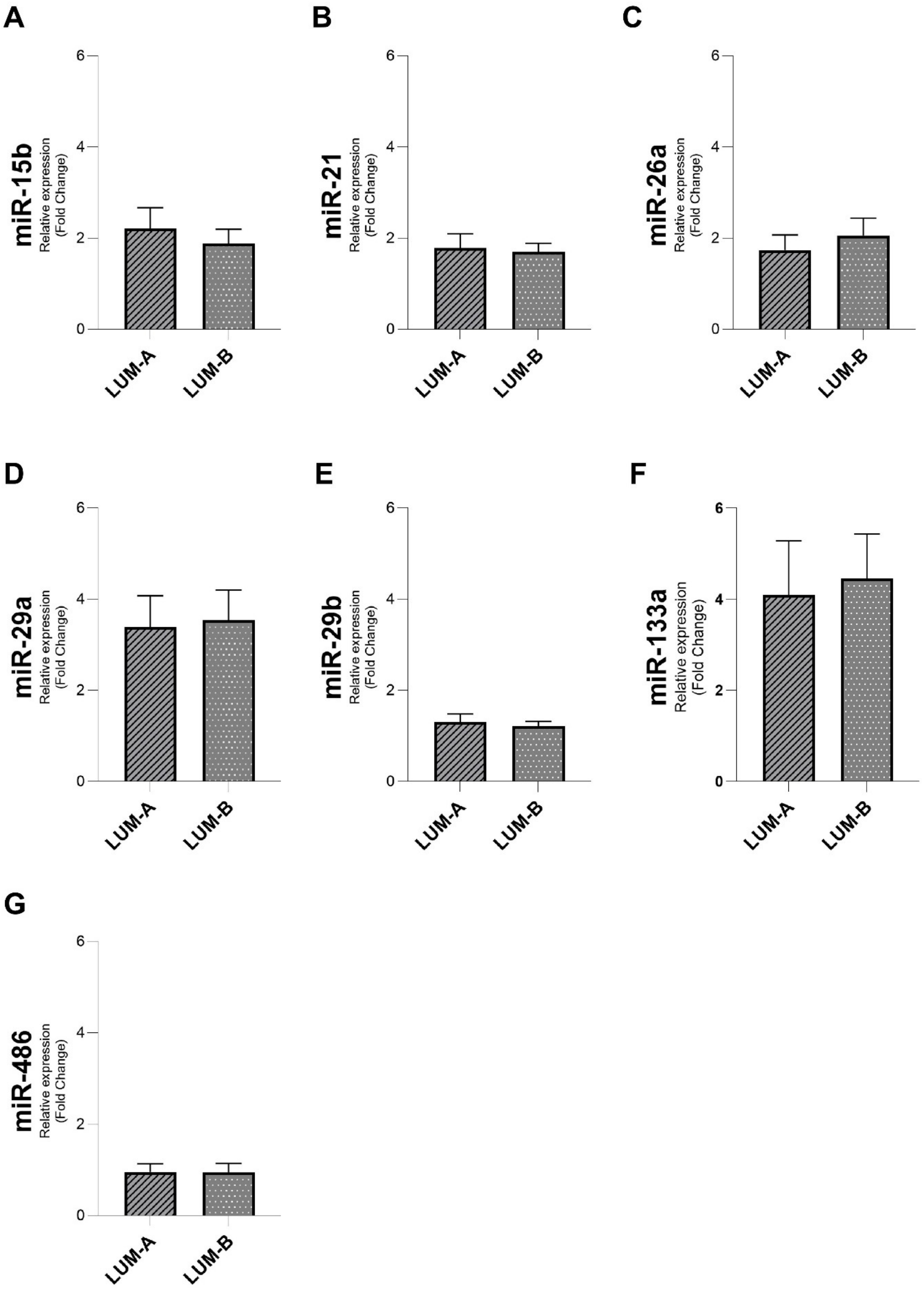

Supplementary Figure S1. Differences in miRNA expression levels in breast cancer patients according to tumor molecular subtypes.

We analyzed miRNA expression levels in BCP with Luminal-A (LUM-A, n=17) or Luminal-B (LUM-B, n=21) molecular subtype. (A)-(G). No significant modulation were observed for the analyzed miRNAs when considering molecular subtype.

Abbreviations: BCP, breast cancer patients; LUM-A, luminal A; LUM-B, luminal B.

Supplementary Figure S2

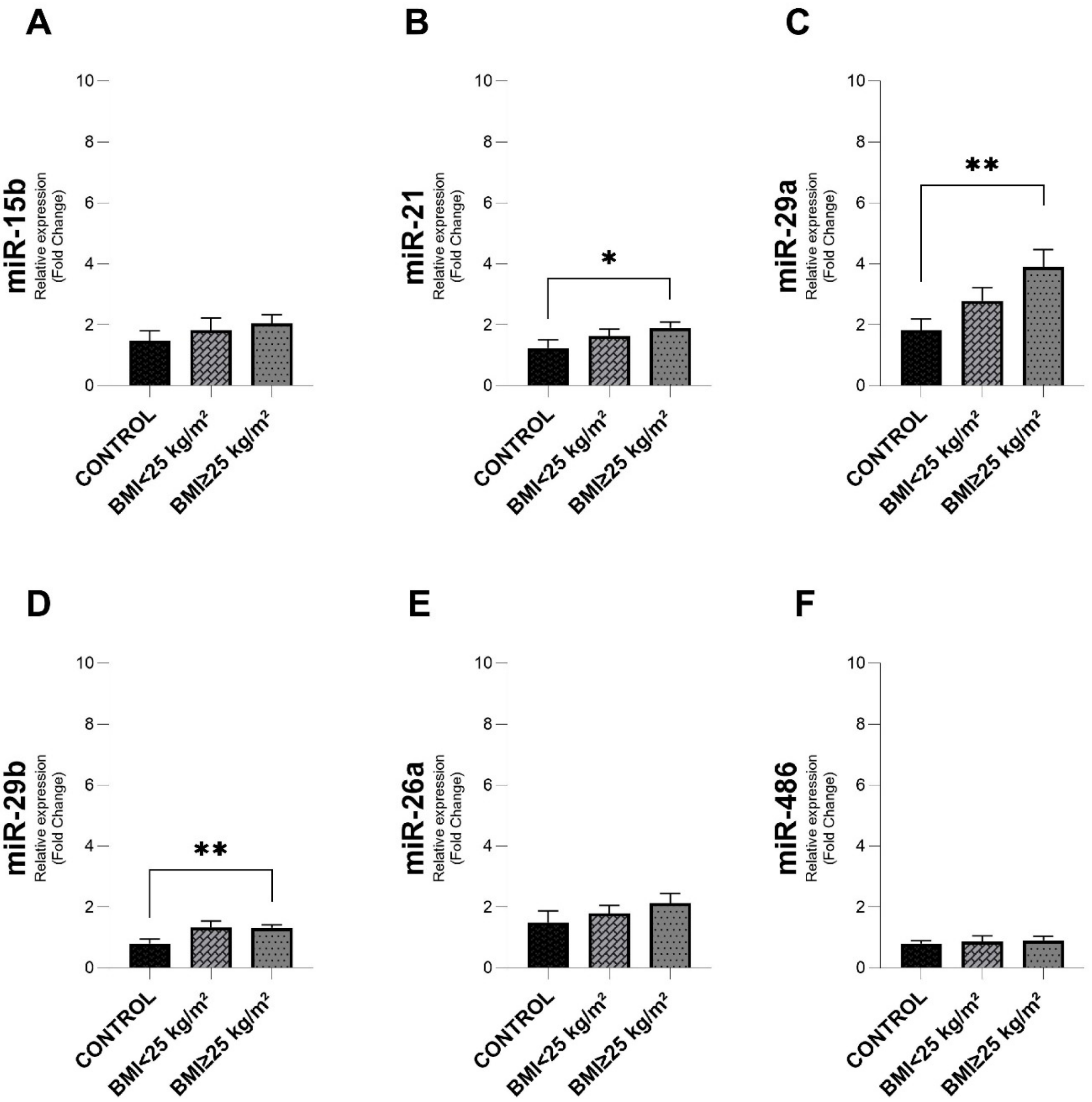

Supplementary Figure S2. Differences in miRNA profile according to body mass index categories.

Circulating miRNA levels were analyzed according to different BMI categories, as follows: BMI < 25 kg/m² (normal weight, n = 15), BMI ≥ 25 kg/m² (overweight and obese, n = 31). Data show an upregulation of miR-21 (p=0.033) (B), miR-29a (p=0.009) (D), and miR-29b (p=0.010) (E) in BCP with BMI ≥ 25 kg/m² compared to controls (n=16). No significant modulation were observed for miR-15b (A), miR-26a (E) and miR-486 (F) when considering BMI categories.

Abbreviations: BCP, breast cancer patients; BMI, body mass index.

Supplementary Figure S3

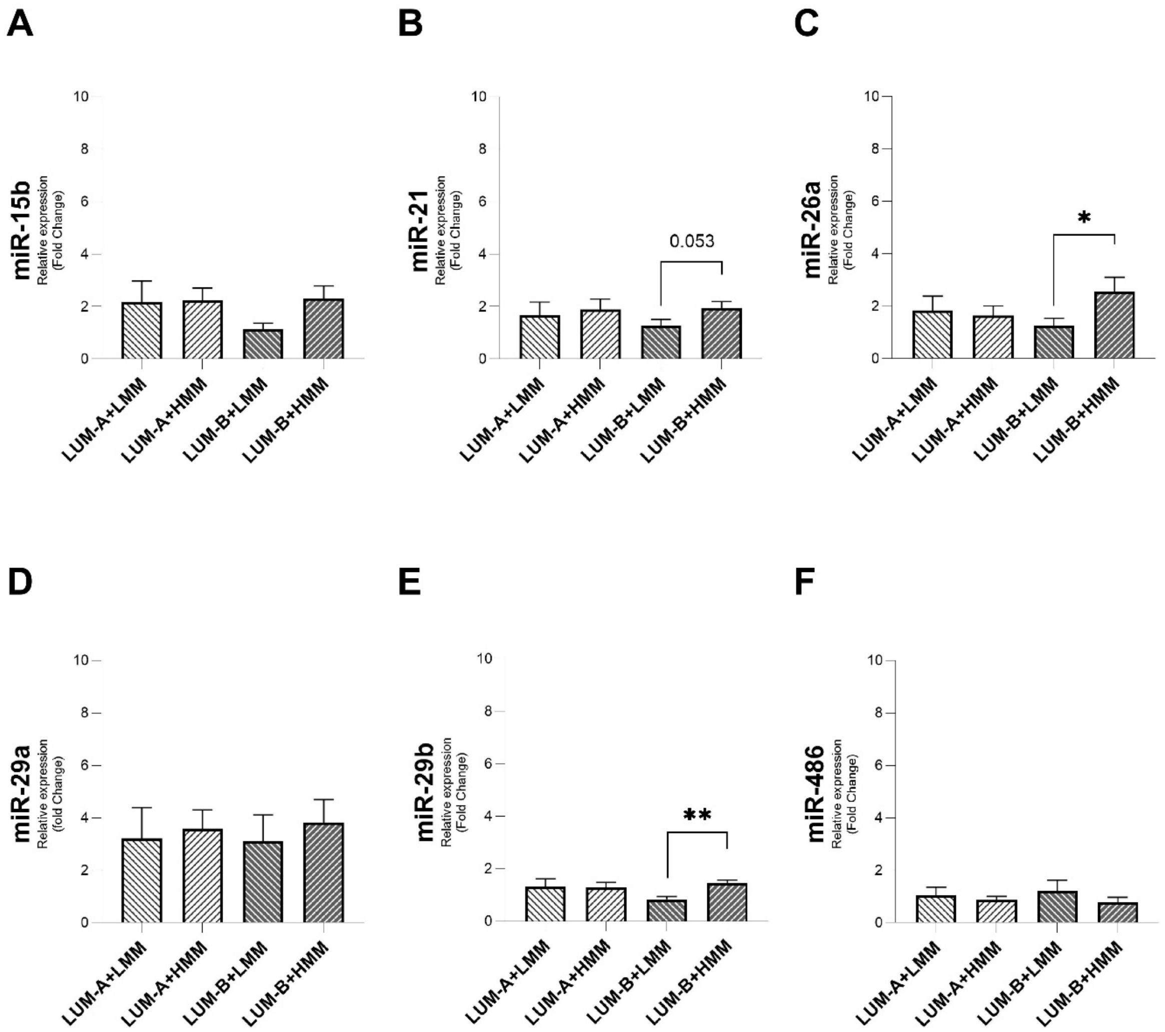

Supplementary Figure S3. Differential expression profile of miRNAs in BCP according to muscularity levels and tumor molecular subtypes.

We analyzed miRNA expression levels in BCP with Lum-A or Lum-B and muscularity level (FFMI median value set at 17 kg/m2 as internal cut-off). Groups were the following: LUM-A + LMM (n=9); LUM-A + HMM (n=8); LUM-B + LMM (n=8); LUM-B + HMM (n=13).

Data shown lower expression levels of miR-26a (p=0.029) (B) and miR-29b (p=0.029) (C) in BCP with LUM-B+LMM vs those LUM-B+HMM. We observed a trend of upregulation of (C) miR-29b in BCP with LUM-A+HMM vs. LUM-B+LMM (p = 0.053), as well as a trend of modulation of (A) miR-21 in LUM-B patients with LMM compared to HMM (p = 0.053). No other significant modulations were observed for the other miRNAs. No other significant modulations were observed for miR-15b (A), miR-29a (D) and miR-486 (F) considering both muscularity levels and tumor molecular subtypes.

Abbreviations: BCP, breast cancer patients; FFMI, fat-free mass index; LUM-A, Luminal-A; LUM-B, Luminal-B; LMM, low muscle mass; HMM, high muscle mass.

Supplementary Figure S4

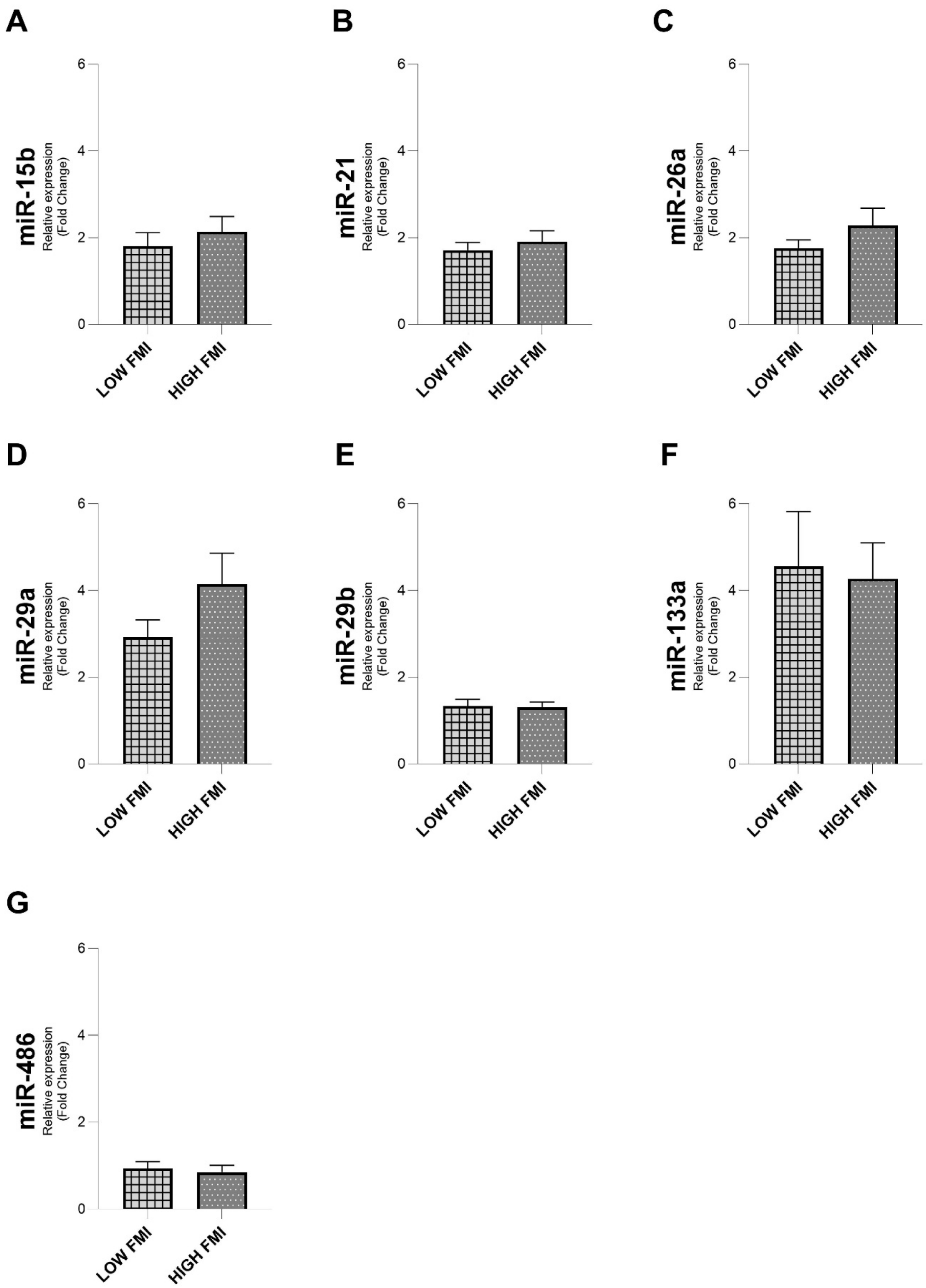

Supplementary Figure S4. Expression profile of circulating miRNAs according to adiposity levels.

Circulating miRNA levels were analyzed considering the median level of FMI (internal cut-off set at 9.9 kg/m²), and BCP were divided into subgroups with low (n=23) and high (n=23) FMI. (A)-(F) No significant modulation of miRNA analyzed were observed when considering adiposity levels in our cohort.

Abbreviations: BCP, breast cancer patients; FMI, fat mass index.
